# Differences in PD‐L1, PD‐L2, and EGFR Expression Between Naive and Recurrent Tumors in Patients With Head and Neck Squamous Cell Carcinoma: A Retrospective Study

**DOI:** 10.1002/hed.28151

**Published:** 2025-03-28

**Authors:** Ryosuke Sato, Takahiro Inoue, Risa Wakisaka, Hiroki Komatsuda, Michihisa Kono, Hidekiyo Yamaki, Kenzo Ohara, Takumi Kumai, Akemi Kosaka, Takayuki Ohkuri, Toshihiro Nagato, Kan Kishibe, Hiroya Kobayashi, Miki Takahara

**Affiliations:** ^1^ Department of Otolaryngology Head & Neck Surgery Asahikawa Medical University Asahikawa Japan; ^2^ Department of Innovative Head & Neck Cancer Research and Treatment Asahikawa Medical University Asahikawa Japan; ^3^ Department of Pathology Asahikawa Medical University Asahikawa Japan

**Keywords:** EGFR, head and neck cancer, immunohistochemistry, PD‐L1, PD‐L2

## Abstract

**Background:**

The efficacy of anti‐PD‐1 and EGFR therapies for head and neck squamous cell carcinoma (HNSCC) can be predicted using various markers; however, the stability of these markers remains unclear.

**Methods:**

In this retrospective study, laboratory findings and tissue expression of PD‐L1, PD‐L2, and EGFR were analyzed in 79 paired naive and recurrent HNSCC tumors. Laboratory findings were also analyzed in nonrecurrent patients using a propensity score‐matched analysis. PD‐L1 and PD‐L2 expression levels were assessed using tumor proportion score (TPS) and combined positive score (CPS), whereas EGFR was evaluated using the H‐score.

**Results:**

White blood cell, neutrophil, lymphocyte, and monocyte counts and lymphocyte‐monocyte ratios were significantly lower in the patients after the first‐line treatment regardless of recurrence. PD‐L1, PD‐L2, and EGFR expression changed in 30%–40% of tumor pairs. Immune but not tumoral PD‐L1 positivity rates were significantly higher in the patients with early recurrence.

**Conclusions:**

The expression of immune checkpoints including PD‐L1 in naive tumors does not reflect those in recurrent tumors. Increasing PD‐L1 expression in immune cells may cause early recurrence of HNSCC.

## 1 Introduction

Programmed death receptor‐1 (PD‐1) inhibitors, such as nivolumab and pembrolizumab, have been shown to improve overall survival (OS) in patients with recurrent or metastatic head and neck squamous cell cancer (R/M HNSCC) in phase 3 clinical trials [1, 2]. As the clinical efficacy of PD‐1 inhibitors varies widely among patients, reliable biomarkers are needed to identify responders. Programmed cell death ligand‐1 (PD‐L1) in tumor tissue is considered a biomarker because HNSCC with positive PD‐L1 expression is associated with improved OS with PD‐1 inhibitors [2]. Programmed cell death ligand‐2 (PD‐L2), another ligand of PD‐1, has also been investigated as a predictor of PD‐1 inhibitors' efficacy in HNSCC [3, 4].

Immune checkpoint expression should be assessed using biopsy specimens from recurrence sites just before administering a PD‐1 inhibitor, as primary tumor specimens may not reflect the current PD‐L1 expression at recurrence. However, obtaining biopsy specimens from recurrence sites is often challenging, especially in cases of distant metastasis, where only samples from past biopsies of primary tumors are available. Most clinical trials of PD‐1 inhibitors have not determined the timing for assessing PD‐L1 expression in tissue specimens [2, 5, 6, 7]. PD‐L1 expression in tumors affects the efficacy of PD‐1 inhibitors; however, little is known about the differences in PD‐L1 expression between naive and recurrent HNSCC.

This study aimed to determine the optimal timing for specimen collection by comparing PD‐L1 and PD‐L2 expression between naive and recurrent tumors, including those with multiple recurrence sites, using paired biopsy specimens from patients with R/M HNSCC. Epidermal growth factor receptor (EGFR) is a representative cancer antigen associated with the progression of HNSCC, for which anti‐EGFR therapies are administered as first‐ or second‐line treatment. EGFR expression levels in naive and recurrent tumors were also investigated.

## 2 Materials and Methods

### 2.1 Study Design and Patients

We retrospectively evaluated differences in the tissue expression levels of PD‐L1, PD‐L2, and EGFR in biopsy specimens obtained at the initial diagnosis and recurrence. Several biomarkers, including immune cells, nutritional status, and inflammation markers, have been associated with the clinical efficacy of PD‐1 inhibitors [8, 9, 10]. Therefore, we also analyzed laboratory findings, including white blood cell counts, neutrophil counts, lymphocyte counts, monocyte counts, neutrophil‐lymphocyte ratios (NLR), lymphocyte‐monocyte ratios (LMR), serum albumin levels, and serum C‐reactive protein (CRP) levels. The study was conducted between January 2012 and December 2022 at Asahikawa medical university hospital.

### 2.2 Ethics

The study adhered to the principles of the Declaration of Helsinki, and written informed consent was obtained from all participants. The study protocol was approved by the Ethics Review Board of Asahikawa Medical University (#16217).

### 2.3 Immunohistochemical Staining

Tissue expression levels were analyzed in formalin‐fixed, paraffin‐embedded biopsy specimens. The rabbit monoclonal Ab clone 28–8 against PD‐L1 (1:400 dilution, ab205921; Abcam), rabbit monoclonal antibody against PD‐L2 (1:200 dilution, #82723; Cell Signaling Technology), and mouse monoclonal antibody against EGFR (1:600 dilution, sc‐373 746; Santa Cruz Biotechnology) served as primary antibodies. The Ventana Benchmark GX (Roche Diagnostics) was used for immunostaining. PD‐L1 and PD‐L2 expression levels were assessed based on positivity, tumor proportion score (TPS), and combined positive score (CPS). In the tumor cells, partial or complete membrane staining at any intensity was considered positive for PD‐L1 or PD‐L2 expression. Immune cells were considered positive for membrane or cytoplasmic staining. The TPS was defined as the total number of positive tumor cells divided by the total number of tumor cells, multiplied by 100. The CPS was defined as the total number of positive cells, including tumor cells and surrounding immune cells (lymphocytes and macrophages), divided by the total number of tumor cells and multiplied by 100. If heterogeneity in PD‐L1 and PD‐L2 expression was observed across different areas, the average value of each area was adopted. Based on previous studies, the positive cutoff points for TPS and CPS were set at ≥ 1% and ≥ 1, respectively [2, 11]. Membrane staining intensity for EGFR was scored as follows: no staining (0), weak (1+), intermediate (2+), and strong (3+). The immunostaining score (H‐score) was calculated, ranging 0–300, using the following formula: 1 × (% of 1 + cells) + 2 × (% of 2+ cells) + 3 × (% of 3+ cells). Positive EGFR expression was defined as an *H* score of ≥ 200.

### 2.4 A Propensity Score‐Matched Analysis

Laboratory findings at each time point (diagnosis of naive tumor, end of the first‐line treatment, and recurrence) between the patients with or without recurrence were investigated. The same number of nonrecurrent patients who matched patient characteristics were selected by a propensity score‐matched analysis. The change rates in immune cells at the end of first‐line treatment and recurrence relative to that at the diagnosis of naive tumor were compared between recurrent and nonrecurrent patients. The immune cell counts in recurrent patients at the time of recurrence were compared with those at 12.5 months after first‐line treatment, which was the median time to recurrence, in nonrecurrent patients.

### 2.5 Statistical Analysis

Patient characteristics were compared between naive and recurrent tumors using Fisher's exact test for nominal variables and one‐way analysis of variance for continuous variables. Differences in laboratory findings and tissue expression of PD‐L1, PD‐L2, and EGFR were analyzed using Wilcoxon signed‐rank tests. The positivity rates for each marker were compared using Fisher's exact test. The differences in the change rates in immune cells between recurrent and nonrecurrent patients were compared using Mann–Whitney *U* tests. Statistical significance was set at *p* < 0.05. EZR (Saitama Medical Center) was used for a propensity score‐matched analysis. All other statistical analyses were performed using GraphPad Prism 10 (GraphPad Software).

## 3 Results

### 3.1 Patient Characteristics

Table 1 summarizes patient characteristics. Among 79 patients whose primary tumor samples were available, 45, 22, and 12 patients with local, lymph node, and lung recurrence, respectively, met the criteria. The median age at diagnosis was 72 (39–90) years. The cohort comprised 68 men and 11 women. The primary sites were as follows: the hypopharynx, 22 (27.8%); oral cavity, 18 (22.8%); oropharynx, 13 (16.5%); larynx, 13 (16.5%); nasopharynx, 4 (5.1%); and other sites, 9 (11.4%). First‐line treatments included surgery alone in 14 patients (17.7%), surgery with adjuvant (chemo)radiotherapy in 5 patients (6.3%), chemoradiotherapy in 50 patients (63.3%), and radiotherapy alone in 10 patients (12.7%). The median interval between diagnosis and recurrence was 12.5 (3.7–192.2) months. No significant differences in patient characteristics were observed between the recurrence modes. Twelve patients (15.2%) were positive for multiple cancers, including esophagus, lung, and colon cancers. One patient with esophageal cancer received chemoradiotherapy, and three patients underwent tumor resection after the treatment of HNSCC.

**TABLE 1:** Patient characteristics.

	Whole patients (*n* = 79)	Local recurrence (*n* = 45)	Lymph node recurrence (*n* = 22)	Lung recurrence (*n* = 12)	*p*
Men:women	68:11	35:10	22:0	11:1	0.07
Age (median)	72 (39–90)	72 (39–90)	70 (52–83)	71 (39–79)	0.95
Primary site					
Hypopharynx	22 (27.8%)	15 (33.3%)	3 (13.6%)	4 (33.3%)	0.62
Oral cavity	18 (22.8%)	11 (24.4%)	7 (31.8%)	0 (0.0%)	
Oropharynx	13 (16.5%)	8 (17.8%)	4 (18.2%)	1 (8.3%)	
Larynx	13 (16.5%)	4 (8.9%)	5 (22.7%)	4 (33.3%)	
Nasopharynx	4 (5.1%)	3 (6.7%)	0 (0.0%)	1 (8.3%)	
Others	9 (11.4%)	4 (8.9%)	3 (13.6%)	2 (16.7%)	
T classifications					
T1	10 (12.7%)	7 (15.6%)	3 (13.6%)	0 (0.0%)	0.88
T2	21 (26.6%)	13 (28.9%)	4 (18.2%)	4 (33.3%)	
T3	15 (19.0%)	6 (13.3%)	6 (27.3%)	3 (25.0%)	
T4	33 (41.8%)	19 (42.2%)	9 (40.9%)	5 (41.7%)	
N classifications					
N0	34 (43.0%)	19 (42.2%)	10 (45.5%)	5 (41.7%)	0.12
N1	13 (16.5%)	6 (13.3%)	4 (18.2%)	3 (25.0%)	
N2	30 (38.0%)	20 (44.4%)	8 (36.4%)	2 (16.7%)	
N3	2 (2.5%)	0 (0.0%)	0 (0.0%)	2 (16.7%)	
M classifications					
M0	79 (100.0%)	45 (100.0%)	22 (100.0%)	12 (100.0%)	NA
M1	0 (0.0%)	0 (0.0%)	0 (0.0%)	0 (0.0%)	
Clinical stages					
I	7 (8.9%)	5 (11.1%)	2 (9.1%)	0 (0.0%)	0.78
II	14 (17.7%)	9 (20.0%)	2 (9.1%)	3 (25.0%)	
III	15 (19.0%)	6 (13.3%)	7 (31.8%)	2 (16.7%)	
IV	43 (54.4%)	25 (55.6%)	11 (50.0%)	7 (58.3%)	
Multiple cancers					
Positive	12 (15.2%)	8 (17.8%)	1 (4.5%)	3 (25.0%)	0.33
Negative	67 (84.8%)	37 (82.2%)	21 (95.5%)	9 (75.0%)	
First‐line treatment					
Surgery	14 (17.7%)	4 (8.9%)	8 (36.4%)	2 (16.7%)	0.37
Surgery with adjuvant (chemo)radiotherapy	5 (6.3%)	3 (6.7%)	2 (9.1%)	0 (0.0%)	
Chemoradiotherapy	50 (63.3%)	31 (68.9%)	11 (50.0%)	8 (66.7%)	
Radiotherapy	10 (12.7%)	7 (15.6%)	1 (4.5%)	2 (16.7%)	
Interval between diagnosis and recurrence (months)	12.5 (3.7–192.2)	12.5 (4.5–192.2)	9.9 (4.0–50.7)	18.8 (3.7–107.7)	0.31

*Note*: Patient characteristics were compared using Fisher's exact test or one‐way ANOVA.

Abbreviation: NA, not applicable.

### 3.2 Laboratory Findings Before and After Recurrence

**FIGURE 1:**
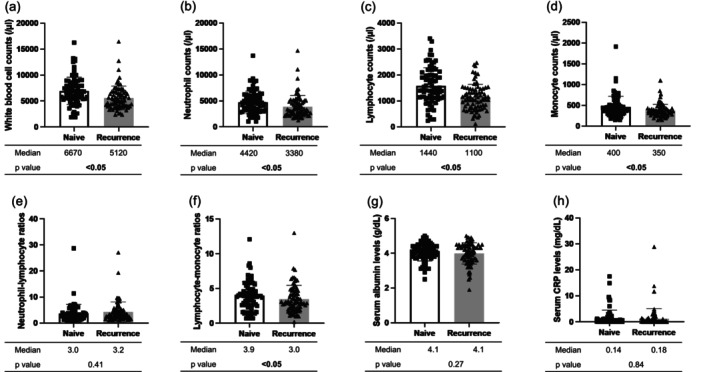
Comparison of laboratory findings between naive and recurrent tumors (79 pairs). (a) White blood cell counts, (b) neutrophil counts, (c) lymphocyte counts, (d) monocyte counts, (e) neutrophil‐lymphocyte ratio, and (f) lymphocyte–monocyte ratios, (g) albumin, (h) CRP were significantly lower in patients with recurrent tumors than in those with naive tumors.

Figure 1 shows the differences in laboratory findings between naive and recurrent tumors in the 79 pairs. White blood cell count, neutrophil count, lymphocyte count, monocyte count, and LMR were significantly lower in patients with recurrent tumors than in those with naive tumors. The median values of these parameters were 6970, 4420, 1440, 400/μL, and 3.9 in naive tumors and 5120, 3380, 1100, 350 μL, and 3.0 in recurrent tumors, respectively. No significant differences were observed in the NLR, serum albumin level, or serum CRP level. The decrease in immune cells was observed only in the patients without multiple cancers; however, the low number of patients with multiple cancers (*n* = 12) might mitigate the significance of the results in the patients with multiple cancers (Figures S1 and S2).

**FIGURE 2:**
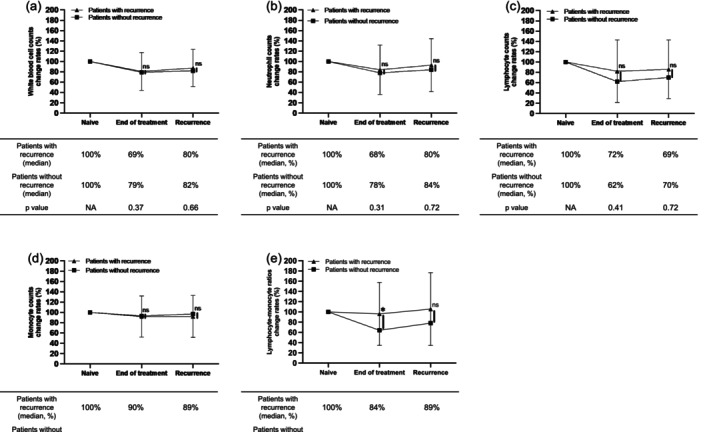
The time course of laboratory findings in the patients with or without recurrence. (a) While blood cell counts, (b) neutrophil counts, (c) lymphocyte counts, (d) monocyte counts, (e) lymphocyte‐monocyte ratios. The figure shows the percentage of immune cells at the end of first‐line treatment and recurrence relative to that at the diagnosis of naive tumor. The immune cell counts in recurrent patients at the time of recurrence were compared with those at 12.5 months after first‐line treatment, which was the median time to recurrence, in nonrecurrent patients. NA, not applicable; ns, not significant.

To investigate whether the decrease of immune cell counts is truly associated with the recurrence, the change rates of immune cells at each time point between recurrent and nonrecurrent patients were analyzed using a propensity score‐matched analysis. No significant differences in patient characteristics were observed between recurrent and nonrecurrent patients (Table S1). As shown in Figure 2, all types of immune cells were decreased at the end of first‐line treatment compared to the initial diagnosis, and had kept decreased regardless of the presence or absence of recurrence. These results indicate that the first‐line treatment but not recurrence would be the cause of decreased immune cells.

### 3.3 Difference in the Immune Checkpoint and EGFR Expressions

First, we investigated the positivity rates for PD‐L1, PD‐L2, and EGFR using paired samples from naive and recurrent tumors. The positivity rates of tumoral and immune PD‐L1 (TI‐PD‐L1), PD‐L2 (TI‐PD‐L2), and EGFR were 58.2%, 34.2%, and 25.3% in naive tumors and 74.7%, 40.5%, and 32.9% in recurrent tumors, respectively (Table 2). The positivity rate of TI‐PD‐L1 in recurrent tumors was significantly higher than that in naive tumors. The expression of tumoral PD‐L1 (T‐PD‐L1) and PD‐L2 (T‐PD‐L2), as well as immune PD‐L1 (I‐PD‐L1) and PD‐L2 (I‐PD‐L2) between naive and recurrent tumors was also examined (Table S2–S5). I‐PD‐L1, but not T‐PD‐L1, showed significantly high expression at recurrence, indicating that PD‐L1 expression was mainly increased in immune cells rather than tumor cells at recurrence. The time to recurrence might be associated with the expression of immune checkpoints. The differences in the expression of each parameter between naive and recurrent tumors categorized by the median time of recurrence (12.5 months) were investigated. Interestingly, immune PD‐L1 expression was significantly upregulated only in the patients with early recurrence, indicating that the increased PD‐L1 expression in immune cells may be involved in early but not late recurrence of HNSCC (Figure S3 and Table S6).

**TABLE 2:** Comparison of tumoral and immune PD‐L1, PD‐L2, and tumoral EGFR positivity rates between naive and recurrent tumors.

	Whole patients		Local recurrence		Lymph node recurrence		Lung recurrence	
Tumoral and immune PD‐L1	Positivity rates	*p*.	Positivity rates	*p*	Positivity rates	*p*	Positivity rates	*p*
Naive	58.2%	**< 0.05**	60.0%	0.26	59.1%	0.33	50.0%	0.40
Recurrent	74.7%		73.3%		77.3%		75.0%	
Tumoral and immune PD‐L2	Positivity rates	*p*	Positivity rates	*p*	Positivity rates	*p*	Positivity rates	*p*
Naive	34.2%	0.51	24.4%	0.64	45.5%	0.36	50.0%	0.68
Recurrent	40.5%		31.1%		63.6%		33.3%	
EGFR	Positivity rates	*p*	Positivity rates	*p*	Positivity rates	*p*	Positivity rates	*p*
Naive	25.3%	0.38	26.7%	0.82	22.7%	0.20	25.0%	> 0.99
Recurrent	32.9%		31.1%		45.5%		16.7%	

*Note*: Positivity rates of each marker were compared using Fisher's exact test. Bold indicates *p* = 0.04.

Abbreviations: EGFR, epidermal growth factor receptor; PD‐L1, programmed cell death ligand‐1; PD‐L2, programmed cell death ligand‐2.

**FIGURE 3:**
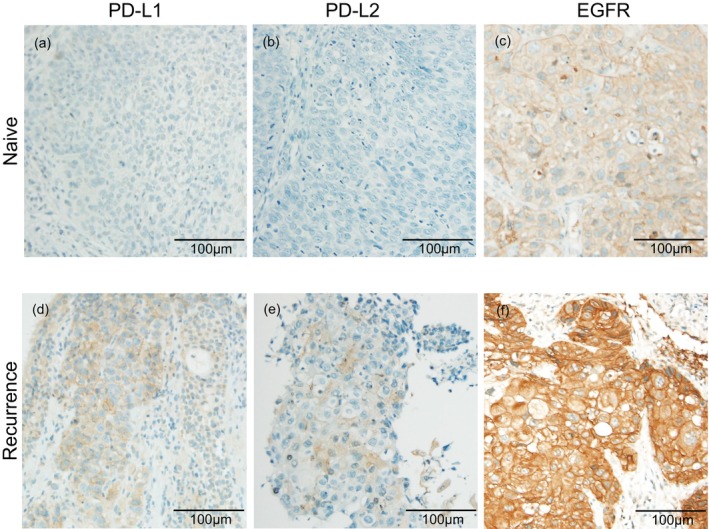
Representative immunohistochemical images of programmed cell death ligand‐1 (PD‐L1), programmed cell death ligand‐2 (PD‐L2), and epidermal growth factor receptor (EGFR) in patients with positive marker conversions at recurrence. (a, d) PD‐L1, (b, e) PD‐L2, (c, f) EGFR. The immunohistochemical images show negative staining of PD‐L1, PD‐L2, and EGFR in naive tumors (a–c), which became positive in recurrent tumors (d–f). The samples in (a),(d), (b),(e), and (c),(f) are derived from the same patients. EGFR, epidermal growth factor receptor; PD‐L1, programmed cell death ligand‐1; PD‐L2, programmed cell death ligand‐2. [Color figure can be viewed at wileyonlinelibrary.com]

TI‐PD‐L2 and EGFR levels were also upregulated during recurrence (Figure 3). The expression levels of TI‐PD‐L1, TI‐PD‐L2, and EGFR were altered in 29 (36.7%), 31 (39.3%), and 30 (38.0%) samples, respectively, upon recurrence compared with that in naive tumors (Table 3). Positive‐to‐negative and negative‐to‐positive conversions for TI‐PD‐L1 occurred in eight (10.1%) and 21 (26.6%) pairs, respectively. For TI‐PD‐L2 and EGFR, positive‐to‐negative and negative‐to‐positive conversions occurred in 13 (16.5%) and 18 (22.8%) pairs, and 12 (15.2%) and 18 (22.8%) pairs, respectively. These results suggest that the expressions of immune checkpoints and essential growth factors are altered with recurrence in > 30% of patients with HNSCC.

**TABLE 3:** Changes in tumoral and immune PD‐L1, PD‐L2, and tumoral EGFR between naive and recurrent tumors.

	No change at recurrence		Changes at recurrence	
	Positive to positive	Negative to negative	Positive to negative	Negative to positive
Tumoral and immune PD‐L1	38 (48.1%)	12 (15.2%)	8 (10.1%)	21 (26.6%)
Tumoral and immune PD‐L2	14 (17.7%)	34 (43.0%)	13 (16.5%)	18 (22.8%)
EGFR	8 (10.1%)	41 (51.9%)	12 (15.2%)	18 (22.8%)

Abbreviations: EGFR, epidermal growth factor receptor; PD‐L1, programmed cell death ligand‐1;PD‐L2, programmed cell death ligand‐2.

**FIGURE 4:**
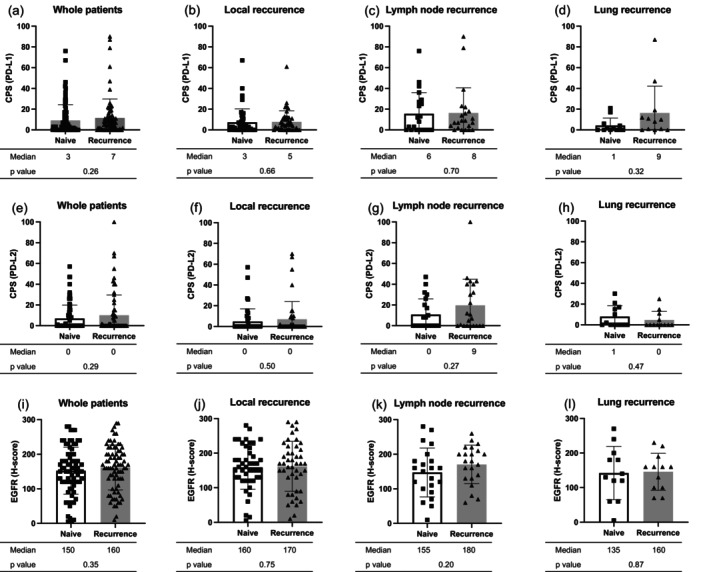
Comparison of PD‐L1 (combined positive score), PD‐L2 (combined positive score), and EGFR (*H* score) expressions between naive and recurrent tumors. (a–d) CPS (PD‐L1) in whole patients, local recurrence, lymph node recurrence, and lung recurrence. (e–h) CPS (PD‐L2) in whole patients, local recurrence, lymph node recurrence, and lung recurrence. (i–l) EGFR in whole patients, local recurrence, lymph node recurrence, and lung recurrence. No significant differences were observed in any marker between naive and recurrent tumors. CPS, combined positive score; EGFR, epidermal growth factor receptor; PD‐L1, programmed cell death ligand‐1; PD‐L2, programmed cell death ligand‐2.

Next, the differences in expression scores instead of positivity for PD‐L1 (CPS), PD‐L2 (CPS), and EGFR (*H* score) between naive and recurrent tumors were examined (Figure 4). The median values of PD‐L1 (CPS), PD‐L2 (CPS), and EGFR (H‐score) in all 79 pairs were 3, 0, and 150 in naive tumors, and 7, 0, and 160 in recurrent tumors. No significant differences were observed in the expression levels of these molecules. Subgroup analysis also revealed no significant differences in PD‐L1 (CPS), PD‐L2 (CPS), or EGFR (H‐score) expression in each mode of recurrence. Similarly, no significant differences were observed in the TPS of PD‐L1 or PD‐L2 between naive and recurrent tumors (Figure S4). Across first‐line treatment types, no significant differences were observed in EGFR, PD‐L1, and PD‐L2 between naive and recurrent tumors except for the expression of EGFR, which was higher in recurrent than in naive tumors only in patients receiving surgery with adjuvant (chemo)radiotherapy (Table S7, Figures S5 and S6).

## 4 Discussion

This study investigated the expression of PD‐L1, PD‐L2, and EGFR in naive and recurrent HNSCC to assess potential markers for predicting treatment response. PD‐L1 expression levels are known to be upregulated in HNSCC cells following chemotherapy such as cisplatin [12] and radiotherapy [13]. We previously demonstrated that gemcitabine enhances PD‐L1 in HNSCC cells [14]. Changes in PD‐L1 expression following treatment have also been investigated in patients with HNSCC. Several studies have reported PD‐L1 overexpression following induction chemotherapy [12, 15, 16, 17] or radiotherapy [13]. However, because PD‐L1 expression in HNSCC cells fluctuates with cell division cycles even in the absence of treatment [18], its role in recurrence remains unclear. Previous studies comparing naive and recurrent tumors have yielded inconsistent results, reporting increased [19], decreased [20], and unchanged [21] PD‐L1 expression levels at HNSCC recurrence. These studies had methodological limitations due to the use of non‐paired tumor specimens, which could not reflect the differences in PD‐L1 expression between naive and recurrent tumors within the same patients. Only two studies with small sample sizes have analyzed paired tumors. One study, which included 33 paired specimens, reported that 36% of HNSCC patients changed their PD‐L1 positivity at recurrence; however, no statistical analysis was conducted [22]. Another study that included 30 paired specimens reported no significant differences in PD‐L1 expression [23]. In other types of cancers, three of four studies using paired specimens reported that PD‐L1 expression increased at recurrence in glioma, ovarian cancer, and craniopharyngiomas [24, 25, 26, 27]. In this study, we examined the expression of PD‐L1, PD‐L2, and EGFR in the largest sample size of paired HNSCC specimens using statistical analysis. The positivity rate of PD‐L1 was consistent with that reported in a previous study [22]. We showed that the positivity rates of tumoral and immune, and immune PD‐L1 were upregulated. Our study also revealed that the positivity of PD‐L1, PD‐L2, or EGFR was altered in 30%–40% of recurrent tumors compared with naive paired tumors. Collectively, the assessment of PD‐L1, PD‐L2, and EGFR expression in naive tumor specimens does not accurately reflect the expression of these molecules at recurrence in patients with HNSCC. In other words, the assessment of PD‐L1 in naive tumors may underestimate its expression in recurrent tumors.

In this study, the positivity rates of immune PD‐L1 were significantly higher in recurrent tumors than in naive tumors. Several types of immune cells express PD‐L1 in addition to tumor [28]. Previous studies showed that macrophages [29], lymphocytes [30, 31], dendritic cells [28], and myeloid cells [32] suppressed the activity of T cells through the PD‐1/PD‐L1 axis. Clinical evidence suggests that counting surrounding immune cells in addition to tumor cells can efficiently predict the response to PD‐1 inhibitors in HNSCC. PD‐L1 (CPS) predicted the efficacy of pembrolizumab in the KEYNOTE‐048 study [2], whereas PD‐L1 (TPS) did not in the CheckMate‐141 study [11]. The superiority of PD‐L1 (CPS) in predicting responders to PD‐1 blockade was also shown in the KEYNOTE‐012 study [33]. These studies suggest the importance of PD‐L1 expression in immune cells in predicting the efficacy of PD‐1 inhibitors in HNSCC. Additionally, our results revealed that the expression of PD‐L1 in immune cells may be important for HNSCC recurrence. We found that only patients with early recurrence showed significantly higher positivity rates of immune PD‐L1, indicating that increased PD‐L1 expression in immune cells may support early recurrence of HNSCC. It is plausible that antitumor immune cells should be suppressed via the PD‐1/PD‐L1 axis at the early phase of recurrence.

PD‐L2 inhibits T cell proliferation, and the binding affinity of PD‐L2 for PD‐1 is three times higher than that of PD‐L1 [34, 35]. High expression of PD‐L2 has been associated with poor prognosis in HNSCC [36]; however, its expression of PD‐L2 has scarcely been evaluated during HNSCC recurrence. EGFR is widely expressed in HNSCC tissues compared with that in normal tissues [37], and its overexpression has been associated with poor OS in HNSCC [38]. Additionally, like PD‐L1, we examined the expression of these molecules in naive and paired recurrent tumors. We discovered no significant differences in PD‐L2 or EGFR expression between naive and recurrent tumors, which is consistent with the findings of previous studies in HNSCC, ovarian cancer, and glioblastoma [23, 39, 40, 41] suggesting that PD‐L1, rather than PD‐L2 or EGFR, would be associated with HNSCC recurrence.

This study has some limitations. First, only cases with available paired specimens of naive and recurrent tumors were included, and examining the time‐course difference in PD‐L1 expression in cured HNSCC was challenging. Whether the upregulated expression of PD‐L1 at recurrence is due to the survival of immune‐resistant tumor clones via selection pressure at the initial treatment or occurs in a time‐dependent manner remains to be elucidated. Second, variations in first‐line treatment may have influenced tissue marker expression and immune cell profiles. Sub‐group analysis of first‐line treatment revealed that the expression of EGFR was high in recurrent tumors in patients who received surgery with adjuvant (chemo)radiotherapy. However, only five patients received this therapy, and further research is necessary to confirm this finding. Third, the expressions of PD‐L1 and PD‐L2 in cancer tissues are known to exhibit heterogeneity. To avoid the selection bias, TPS and CPS scores were assessed by counting the average value of multiple areas for objective results. Nevertheless, it is difficult to predict whether the results of biopsy samples can represent the biological features of the whole tumor. Fourth, HNSCC is known to have a high frequency of multiple cancers. Although a subgroup analysis divided by the presence of multiple cancers showed that the changes in immune cells were only evident in the patients without multiple cancers, only 12 patients had multiple cancers, which may mitigate the statistical significance of the results. Further study with a large number of patients is required to confirm whether the presence of multiple cancers affects the immunological parameters of HNSCC.

In conclusion, this study demonstrated that PD‐L1, PD‐L2, and EGFR expression changed in 30%–40% of recurrent tumors, suggesting that marker expression in naive tumor specimens may not accurately reflect their status at recurrence. Additionally, recurrent tumors exhibited significantly higher positivity rates for immune PD‐L1 in early recurrence, indicating that the early recurrence of HNSCC is mediated through immune regulation by upregulated PD‐L1, which would be an ideal target for the PD‐1/PD‐L1 inhibitors.

## Author Contributions

**Ryosuke Sato:** data curation, formal analysis, and investigation, methodology, writing – original draft. **Takahiro Inoue:** data curation, formal analysis, and investigation. **Risa Wakisaka:** data curation, formal analysis, and investigation. **Hiroki Komatsuda:** data curation, formal analysis, and investigation. **Michihisa Kono:** data curation, formal analysis, and investigation. **Hidekiyo Yamaki:** data curation, formal analysis, and investigation. **Kenzo Ohara:** data curation, formal analysis, and investigation. **Takumi Kumai:** data curation, formal analysis, and investigation, methodology, conceptualization and supervision, writing – original draft, writing – review and editing. **Akemi Kosaka:** data curation, formal analysis, and investigation. **Takayuki Ohkuri:** data curation, formal analysis, and investigation. **Toshihiro Nagato:** data curation, formal analysis, and investigation. **Kan Kishibe:** data curation, formal analysis, and investigation. **Hiroya Kobayashi:** data curation, formal analysis, and investigation, writing – review and editing. **Miki Takahara:** data curation, formal analysis, and investigation.

## Ethics Statement

All experiments were approved by the Institutional Ethics Committee of Asahikawa Medical University (#16217). This study was conducted in accordance with the principles of the Declaration of Helsinki.

## Consent

Written informed consent was obtained from all the participants.

## Conflicts of Interest

The authors declare no conflicts of interest.

## Supporting information


**Figure S1.** Comparison of laboratory findings between naive and recurrent tumors in patients with multiple cancers. No significant differences were observed between naive and recurrent tumors.
**Figure S2**. Comparison of laboratory findings between naive and recurrent tumors in patients without multiple cancers. (a) White blood cell counts, (b) neutrophil counts, (c) lymphocyte counts, (d) monocyte counts, and (f) lymphocyte–monocyte ratios were significantly lower in patients with recurrent tumors than in those with naive tumors.
**Figure S3**. Comparison of PD‐L1 (tumor proportion score and CPS), PD‐L2 (tumor proportion score and CPS), and EGFR (*H* score) between naive and recurrent tumors categorized by median time of recurrence. The expression of each marker was categorized by median time of recurrence (12.5 months). No significant differences were observed between naive and recurrent tumors in both early and late of recurrences. CPS, combined positive score; EGFR, epidermal growth factor receptor; M, months; PD‐L1, programmed cell death ligand‐1; PD‐L2, programmed cell death ligand‐2; TPS, tumor proportion score.
**Figure S4**. Comparison of PD‐L1 (tumor proportion score) and PD‐L2 (tumor proportion score) between naive and recurrent tumors at each recurrence site. No significant differences were observed in each marker between naive and recurrent tumors. PD‐L1, programmed cell death ligand‐1; PD‐L2, programmed cell death ligand‐2; TPS, tumor proportion score.
**Figure S5**. Comparison of PD‐L1 (CPS), PD‐L2 (CPS), and EGFR (H‐score) expressions between naive and recurrent tumors categorized by the first‐line treatment. The expression in EGFR was significantly higher in recurrent than naive tumors in patients received surgery with adjuvant (chemo)radiotherapy. No other significant differences were observed in any markers between naive and recurrent tumors. CPS, combined positive score; EGFR, epidermal growth factor receptor; PD‐L1, programmed cell death ligand‐1; PD‐L2, programmed cell death ligand‐2.
**Figure S6**. Comparison of PD‐L1 (tumor proportion score) and PD‐L2 (tumor proportion score) between naive and recurrent tumors categorized by the first line treatment. No significant differences were observed between naive and recurrent tumors in any types of treatment. PD‐L1, programmed cell death ligand‐1; PD‐L2, programmed cell death ligand‐2; TPS, tumor proportion score.

**Tables S1–S7.**Supporting Information.

## Data Availability

The data that support the findings of this study are available on request from the corresponding author. The data are not publicly available due to privacy or ethical restrictions.
