# Supplementary material for: Differences in PD‐L1, PD‐L2, and EGFR Expression Between Naive and Recurrent Tumors in Patients With Head and Neck Squamous Cell Carcinoma: A Retrospective Study
Source: Head Neck. 2025 Mar 28;47(8):2260–9. doi: 10.1002/hed.28151 (PMC12248277; doi:10.1002/hed.28151)
Supplement: Supplementary file 1 — Figure S1. Comparison of laboratory findings between naive and recurrent tumors in patients with multiple cancers. No significant differences were observed between naive and recurrent tumors. Figure S2. Comparison of laboratory findings between naive and recurrent tumors in patients without multiple cancers. (a) White blood cell counts, (b) neutrophil counts, (c) lymphocyte counts, (d) monocyte counts, and (f) lymphocyte–monocyte ratios were significantly lower in patients with recurrent tumors than in those with naive tumors. Figure S3. Comparison of PD‐L1 (tumor proportion score and CPS), PD‐L2 (tumor proportion score and CPS), and EGFR (H score) between naive and recurrent tumors categorized by median time of recurrence. The expression of each marker was categorized by median time of recurrence (12.5 months). No significant differences were observed between naive and recurrent tumors in both early and late of recurrences. CPS, combined positive score; EGFR, epidermal growth factor receptor; M, months; PD‐L1, programmed cell death ligand‐1; PD‐L2, programmed cell death ligand‐2; TPS, tumor proportion score. Figure S4. Comparison of PD‐L1 (tumor proportion score) and PD‐L2 (tumor proportion score) between naive and recurrent tumors at each recurrence site. No significant differences were observed in each marker between naive and recurrent tumors. PD‐L1, programmed cell death ligand‐1; PD‐L2, programmed cell death ligand‐2; TPS, tumor proportion score. Figure S5. Comparison of PD‐L1 (CPS), PD‐L2 (CPS), and EGFR (H‐score) expressions between naive and recurrent tumors categorized by the first‐line treatment. The expression in EGFR was significantly higher in recurrent than naive tumors in patients received surgery with adjuvant (chemo)radiotherapy. No other significant differences were observed in any markers between naive and recurrent tumors. CPS, combined positive score; EGFR, epidermal growth factor receptor; PD‐L1, programmed cell death ligand‐1; PD‐L2, programm [file HED-47-2260-s001.pptx]

## Slide 1
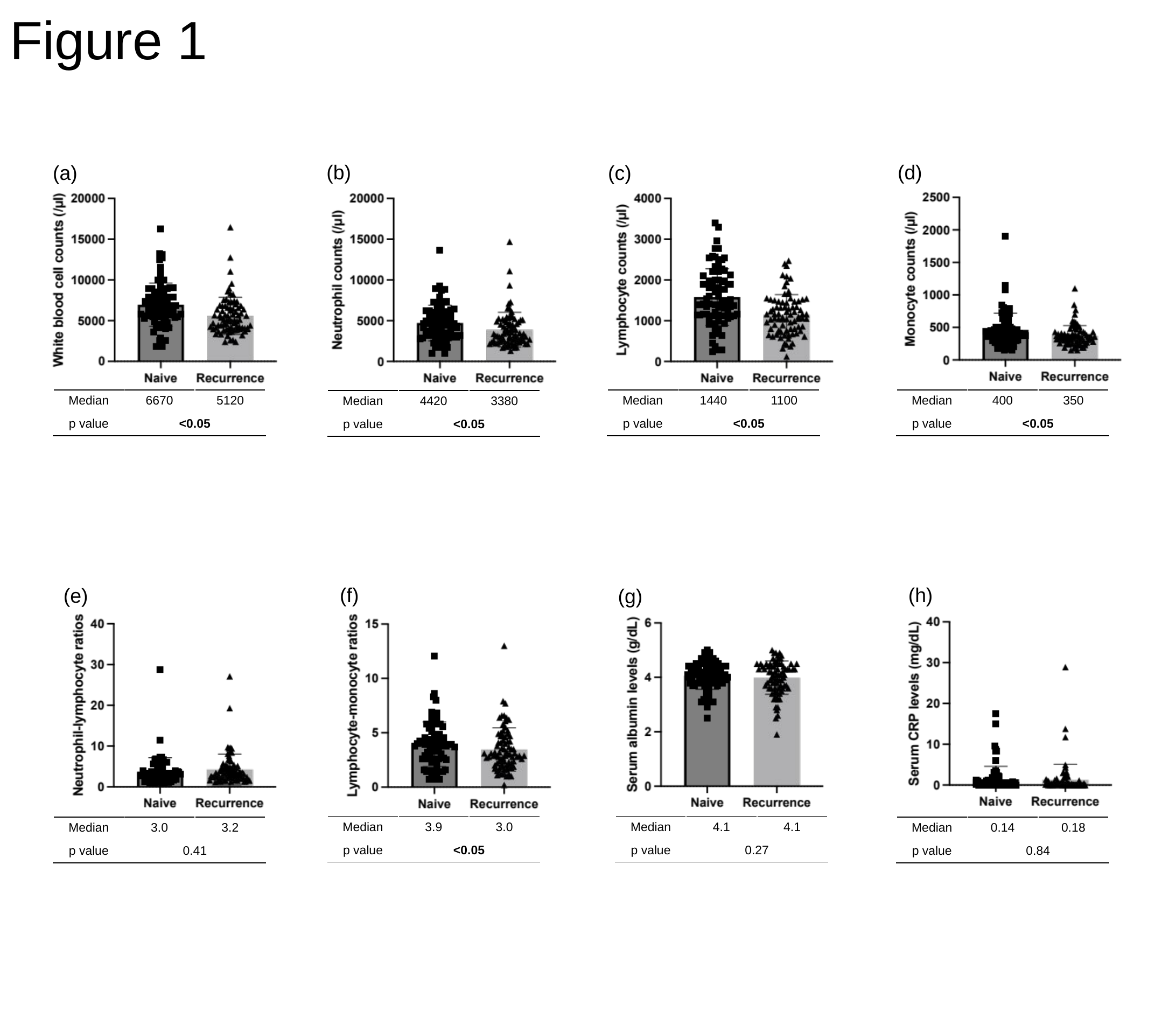

Figure 1
(b)
(d)
(a)
(c)
| Median | 6670 | 5120 |
| --- | --- | --- |
| p value | <0.05 | |
| Median | 1440 | 1100 |
| --- | --- | --- |
| p value | <0.05 | |
| Median | 400 | 350 |
| --- | --- | --- |
| p value | <0.05 | |
| Median | 4420 | 3380 |
| --- | --- | --- |
| p value | <0.05 | |
(f)
(h)
(e)
(g)
| Median | 3.9 | 3.0 |
| --- | --- | --- |
| p value | <0.05 | |
| Median | 4.1 | 4.1 |
| --- | --- | --- |
| p value | 0.27 | |
| Median | 3.0 | 3.2 |
| --- | --- | --- |
| p value | 0.41 | |
| Median | 0.14 | 0.18 |
| --- | --- | --- |
| p value | 0.84 | |

## Slide 2
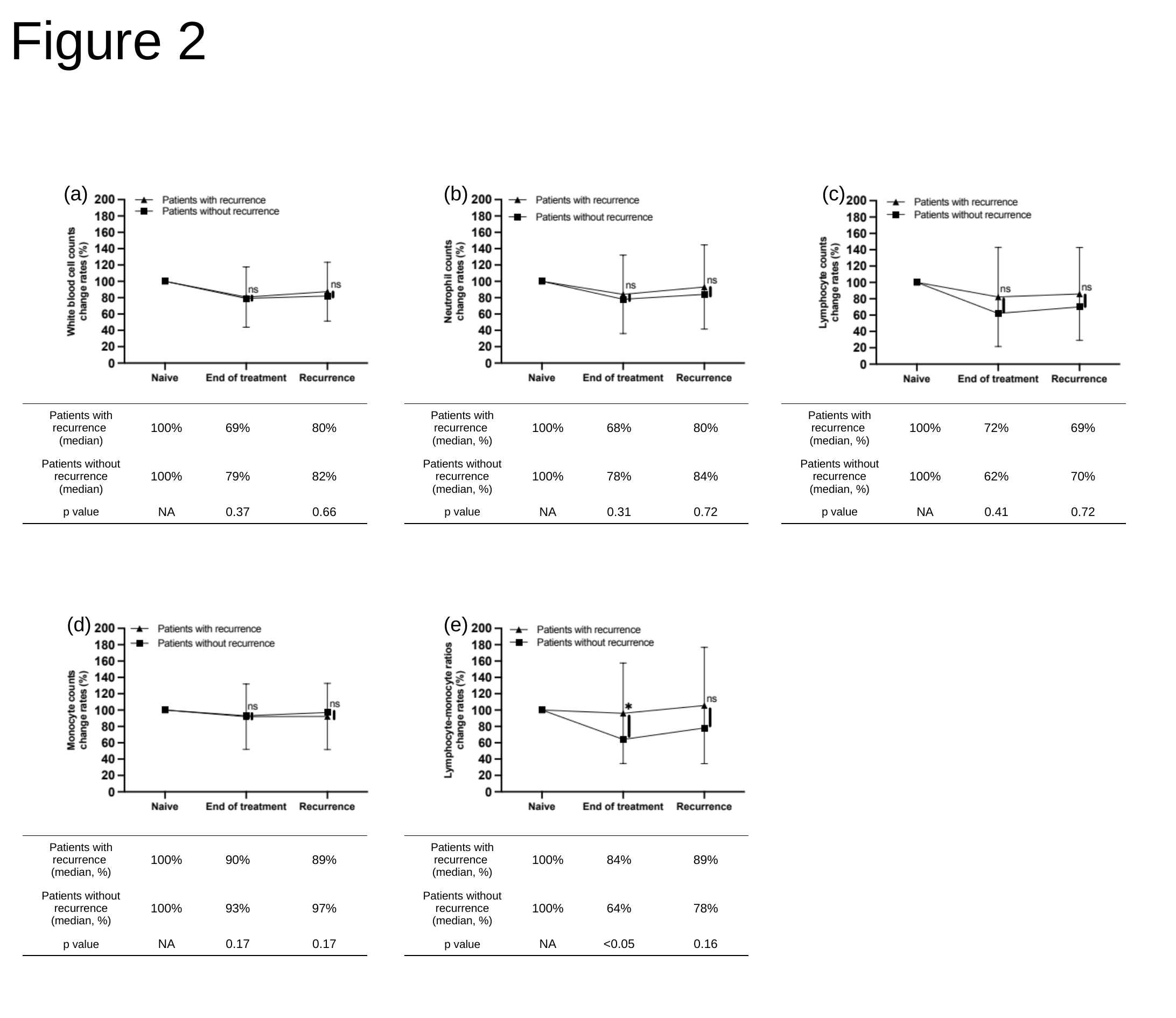

Figure 2
(a)
(b)
(c)
| Patients with recurrence (median) | 100% | 69% | 80% |
| --- | --- | --- | --- |
| Patients without recurrence (median) | 100% | 79% | 82% |
| p value | NA | 0.37 | 0.66 |
| Patients with recurrence (median, %) | 100% | 68% | 80% |
| --- | --- | --- | --- |
| Patients without recurrence (median, %) | 100% | 78% | 84% |
| p value | NA | 0.31 | 0.72 |
| Patients with recurrence (median, %) | 100% | 72% | 69% |
| --- | --- | --- | --- |
| Patients without recurrence (median, %) | 100% | 62% | 70% |
| p value | NA | 0.41 | 0.72 |
(d)
(e)
| Patients with recurrence (median, %) | 100% | 90% | 89% |
| --- | --- | --- | --- |
| Patients without recurrence (median, %) | 100% | 93% | 97% |
| p value | NA | 0.17 | 0.17 |
| Patients with recurrence (median, %) | 100% | 84% | 89% |
| --- | --- | --- | --- |
| Patients without recurrence (median, %) | 100% | 64% | 78% |
| p value | NA | <0.05 | 0.16 |

## Slide 3
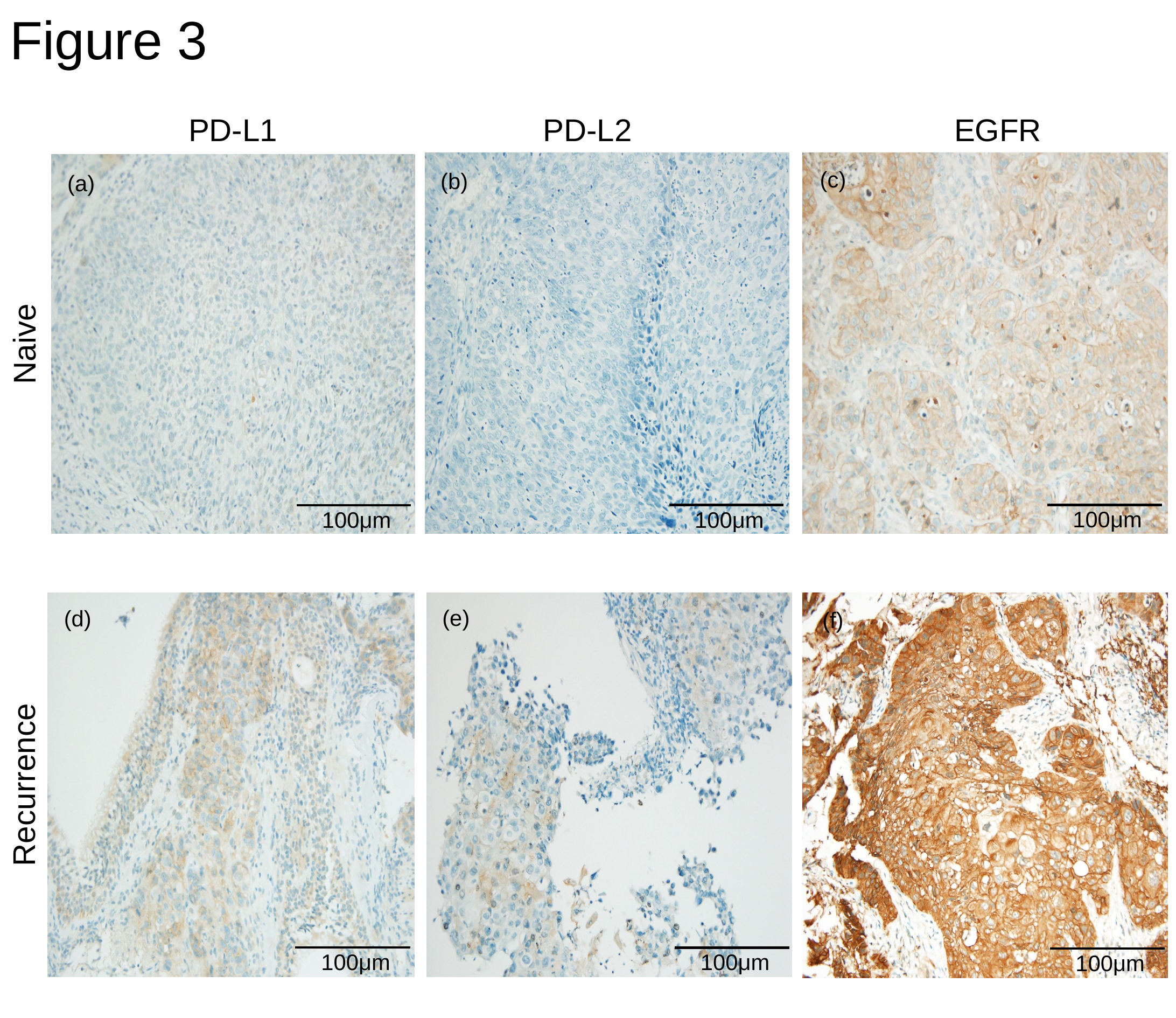

Figure 3
PD-L1
PD-L2
EGFR
(c)
(b)
(a)
Naive
100μm
100μm
100μm
(e)
(d)
(f)
Recurrence
100μm
100μm
100μm

## Slide 4
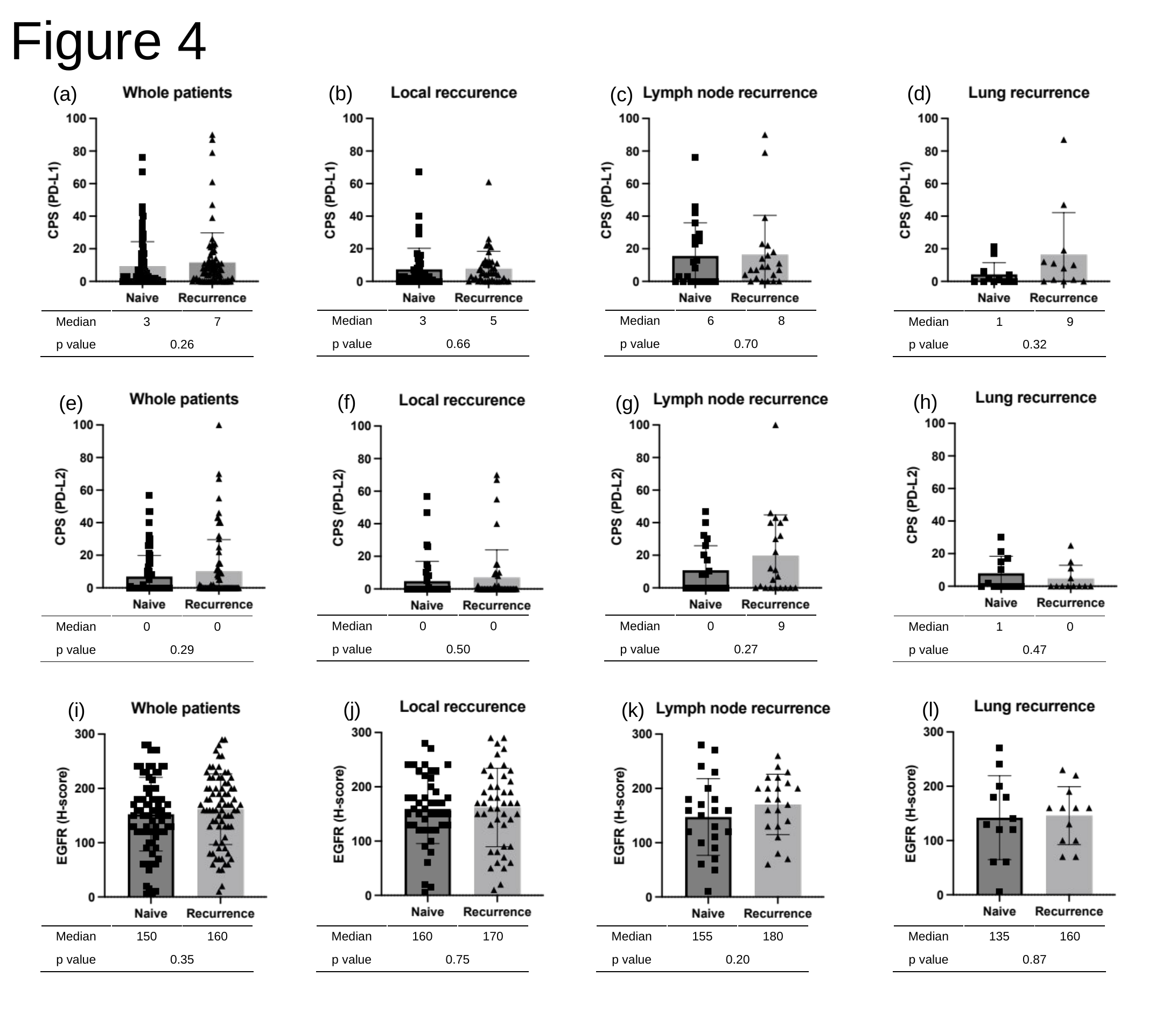

Figure 4
(b)
(d)
(a)
(c)
| Median | 3 | 5 |
| --- | --- | --- |
| p value | 0.66 | |
| Median | 6 | 8 |
| --- | --- | --- |
| p value | 0.70 | |
| Median | 3 | 7 |
| --- | --- | --- |
| p value | 0.26 | |
| Median | 1 | 9 |
| --- | --- | --- |
| p value | 0.32 | |
(f)
(h)
(e)
(g)
| Median | 0 | 0 |
| --- | --- | --- |
| p value | 0.50 | |
| Median | 0 | 9 |
| --- | --- | --- |
| p value | 0.27 | |
| Median | 0 | 0 |
| --- | --- | --- |
| p value | 0.29 | |
| Median | 1 | 0 |
| --- | --- | --- |
| p value | 0.47 | |
(j)
(l)
(i)
(k)
| Median | 150 | 160 |
| --- | --- | --- |
| p value | 0.35 | |
| Median | 160 | 170 |
| --- | --- | --- |
| p value | 0.75 | |
| Median | 155 | 180 |
| --- | --- | --- |
| p value | 0.20 | |
| Median | 135 | 160 |
| --- | --- | --- |
| p value | 0.87 | |

## Slide 5
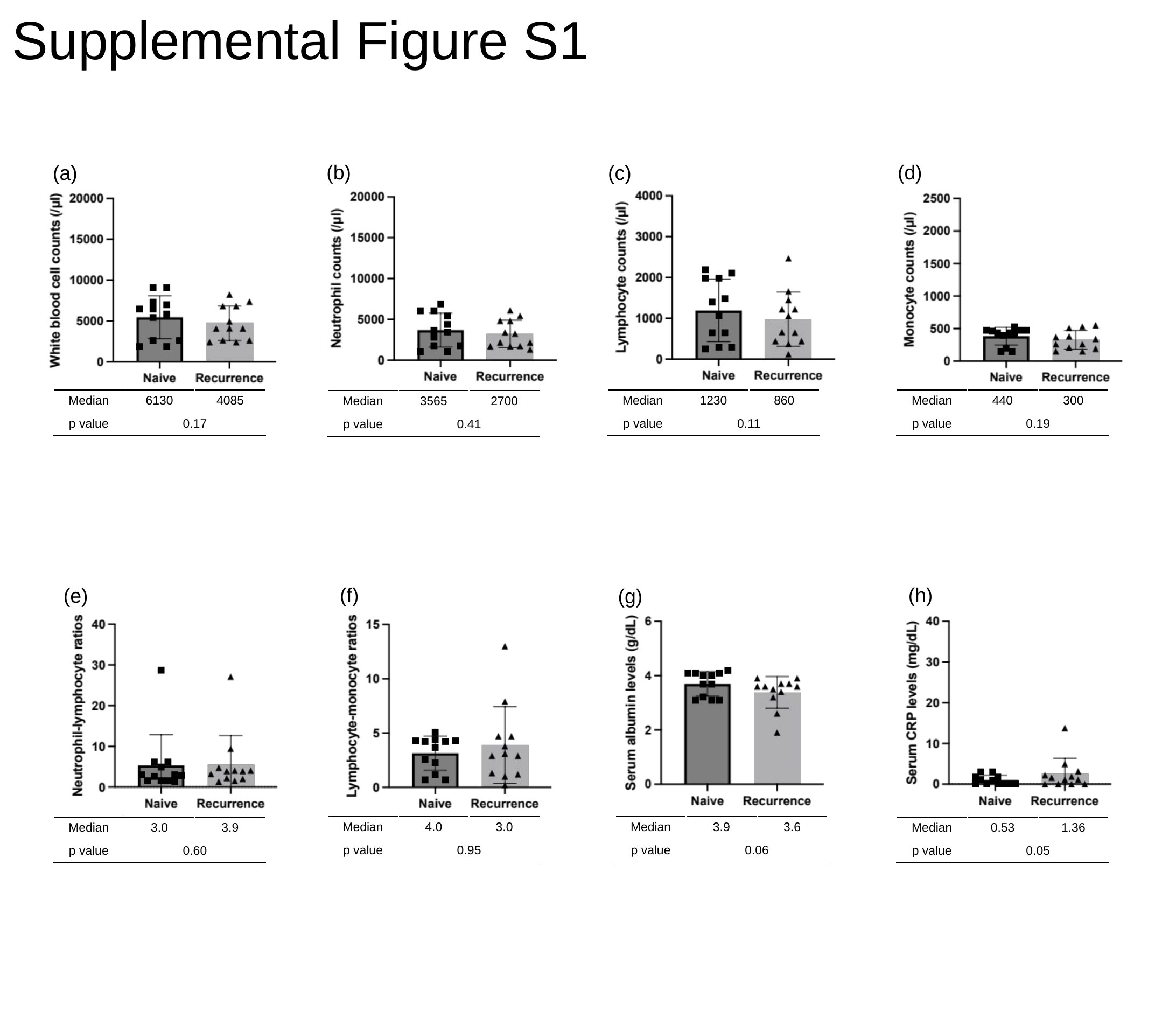

Supplemental Figure S1
(b)
(d)
(a)
(c)
| Median | 6130 | 4085 |
| --- | --- | --- |
| p value | 0.17 | |
| Median | 1230 | 860 |
| --- | --- | --- |
| p value | 0.11 | |
| Median | 440 | 300 |
| --- | --- | --- |
| p value | 0.19 | |
| Median | 3565 | 2700 |
| --- | --- | --- |
| p value | 0.41 | |
(f)
(h)
(e)
(g)
| Median | 4.0 | 3.0 |
| --- | --- | --- |
| p value | 0.95 | |
| Median | 3.9 | 3.6 |
| --- | --- | --- |
| p value | 0.06 | |
| Median | 3.0 | 3.9 |
| --- | --- | --- |
| p value | 0.60 | |
| Median | 0.53 | 1.36 |
| --- | --- | --- |
| p value | 0.05 | |

## Slide 6
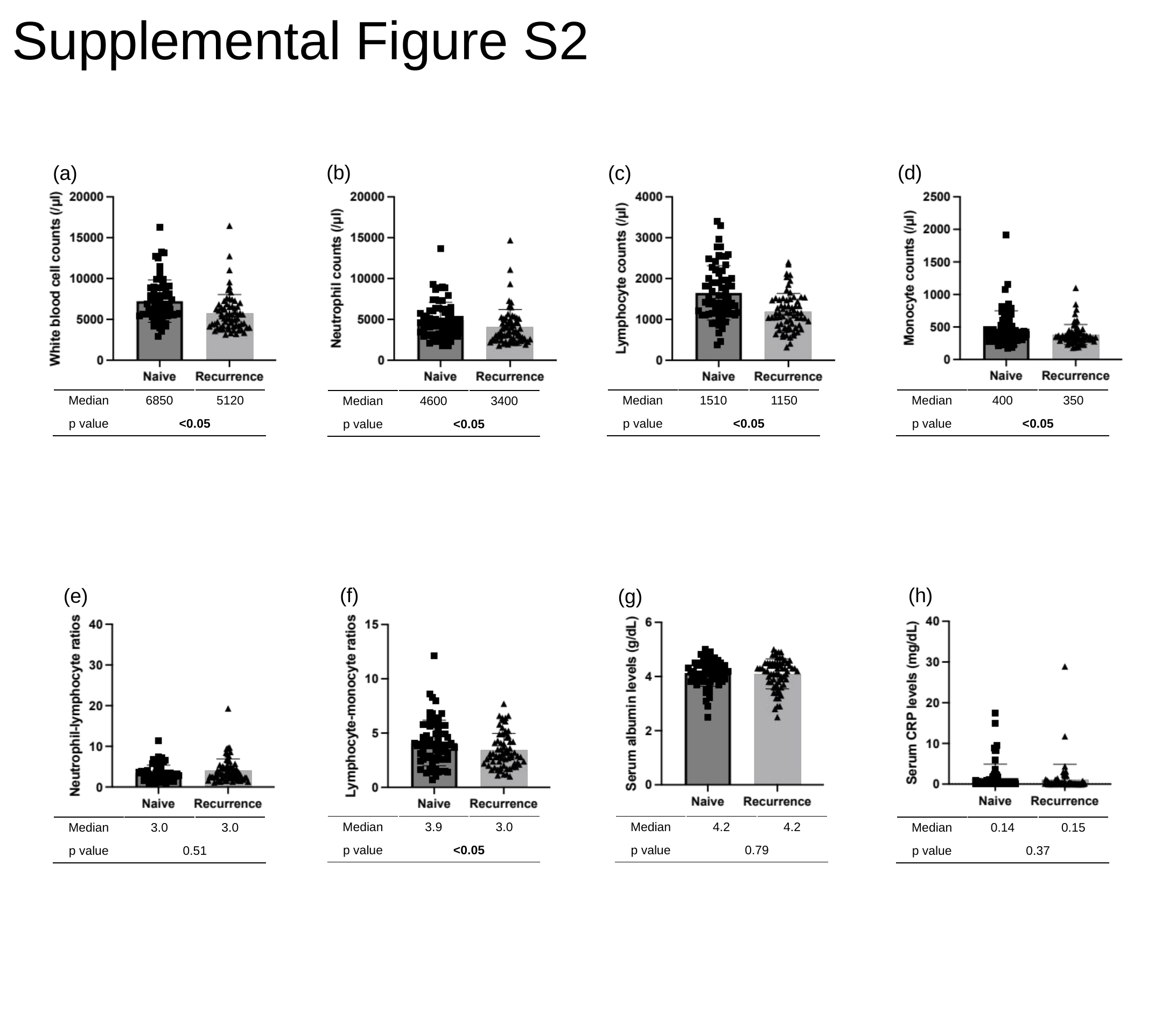

Supplemental Figure S2
(b)
(d)
(a)
(c)
| Median | 6850 | 5120 |
| --- | --- | --- |
| p value | <0.05 | |
| Median | 1510 | 1150 |
| --- | --- | --- |
| p value | <0.05 | |
| Median | 400 | 350 |
| --- | --- | --- |
| p value | <0.05 | |
| Median | 4600 | 3400 |
| --- | --- | --- |
| p value | <0.05 | |
(f)
(h)
(e)
(g)
| Median | 3.9 | 3.0 |
| --- | --- | --- |
| p value | <0.05 | |
| Median | 4.2 | 4.2 |
| --- | --- | --- |
| p value | 0.79 | |
| Median | 3.0 | 3.0 |
| --- | --- | --- |
| p value | 0.51 | |
| Median | 0.14 | 0.15 |
| --- | --- | --- |
| p value | 0.37 | |

## Slide 7
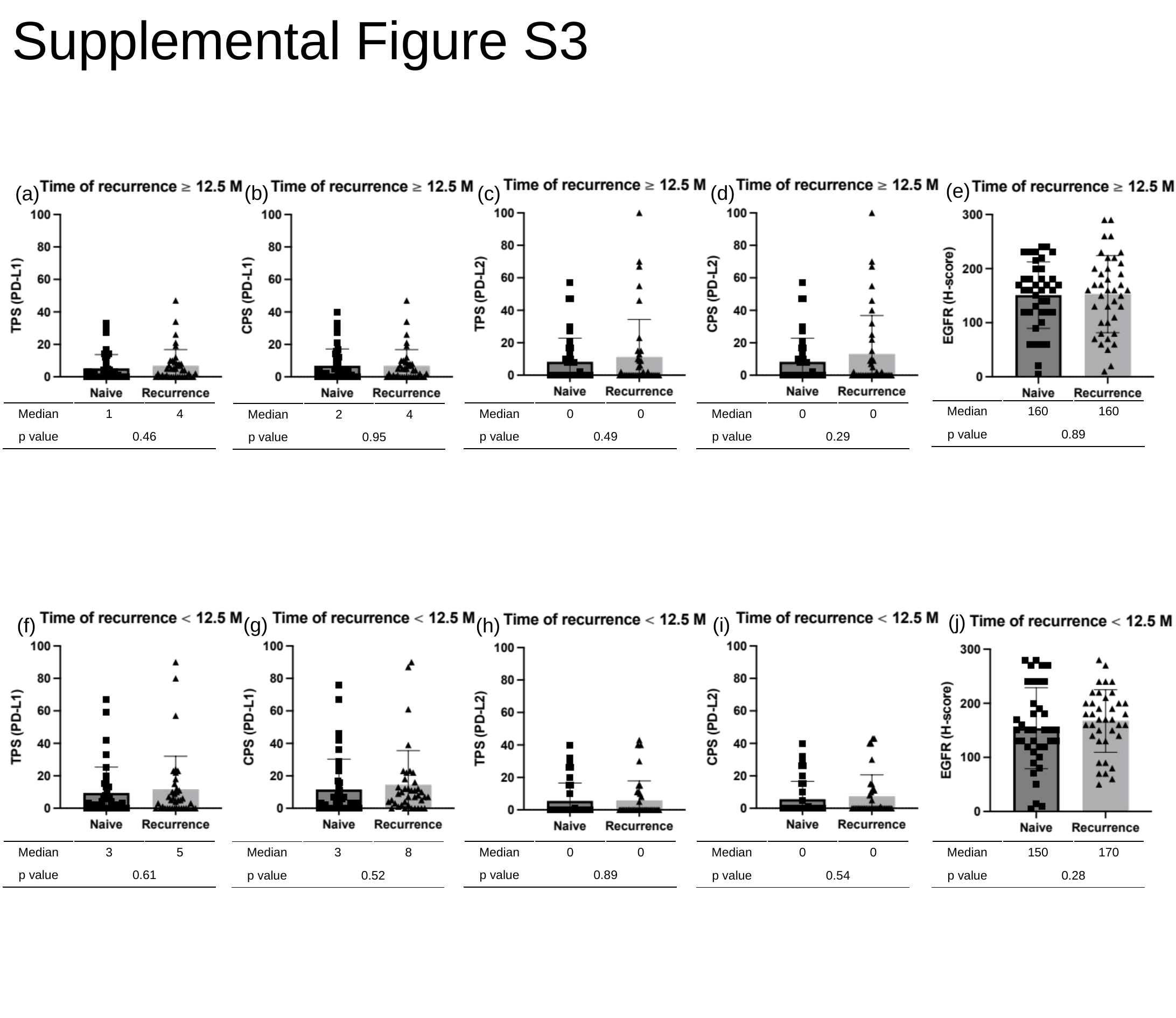

Supplemental Figure S3
(e)
(b)
(d)
(a)
(c)
| Median | 160 | 160 |
| --- | --- | --- |
| p value | 0.89 | |
| Median | 1 | 4 |
| --- | --- | --- |
| p value | 0.46 | |
| Median | 0 | 0 |
| --- | --- | --- |
| p value | 0.49 | |
| Median | 0 | 0 |
| --- | --- | --- |
| p value | 0.29 | |
| Median | 2 | 4 |
| --- | --- | --- |
| p value | 0.95 | |
(j)
(g)
(i)
(f)
(h)
| Median | 3 | 5 |
| --- | --- | --- |
| p value | 0.61 | |
| Median | 0 | 0 |
| --- | --- | --- |
| p value | 0.89 | |
| Median | 0 | 0 |
| --- | --- | --- |
| p value | 0.54 | |
| Median | 150 | 170 |
| --- | --- | --- |
| p value | 0.28 | |
| Median | 3 | 8 |
| --- | --- | --- |
| p value | 0.52 | |

## Slide 8
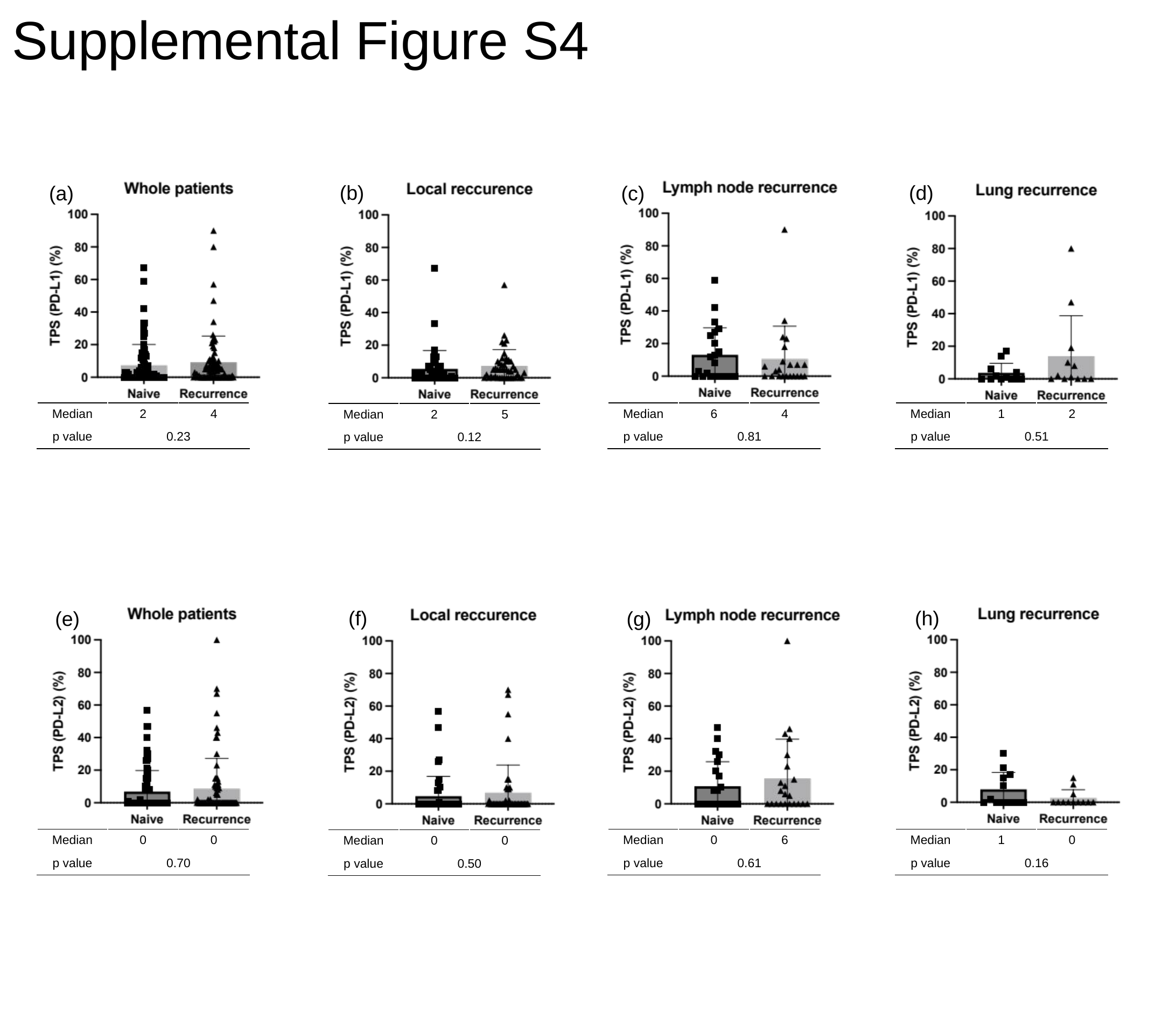

Supplemental Figure S4
(b)
(d)
(a)
(c)
| Median | 2 | 4 |
| --- | --- | --- |
| p value | 0.23 | |
| Median | 6 | 4 |
| --- | --- | --- |
| p value | 0.81 | |
| Median | 1 | 2 |
| --- | --- | --- |
| p value | 0.51 | |
| Median | 2 | 5 |
| --- | --- | --- |
| p value | 0.12 | |
(f)
(h)
(e)
(g)
| Median | 0 | 0 |
| --- | --- | --- |
| p value | 0.70 | |
| Median | 0 | 6 |
| --- | --- | --- |
| p value | 0.61 | |
| Median | 1 | 0 |
| --- | --- | --- |
| p value | 0.16 | |
| Median | 0 | 0 |
| --- | --- | --- |
| p value | 0.50 | |

## Slide 9
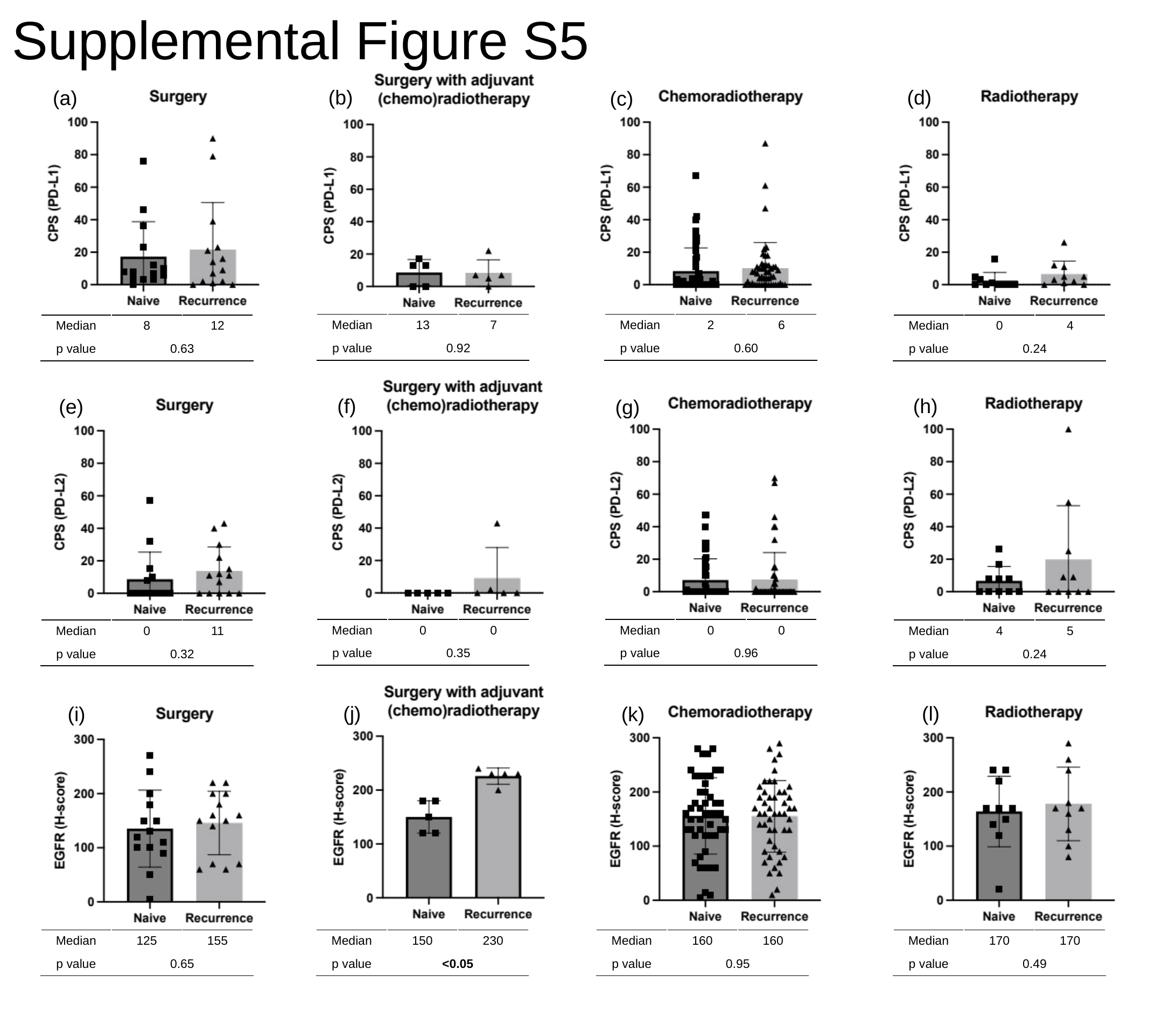

Supplemental Figure S5
(b)
(d)
(a)
(c)
| Median | 13 | 7 |
| --- | --- | --- |
| p value | 0.92 | |
| Median | 2 | 6 |
| --- | --- | --- |
| p value | 0.60 | |
| Median | 8 | 12 |
| --- | --- | --- |
| p value | 0.63 | |
| Median | 0 | 4 |
| --- | --- | --- |
| p value | 0.24 | |
(f)
(h)
(e)
(g)
| Median | 0 | 0 |
| --- | --- | --- |
| p value | 0.35 | |
| Median | 0 | 0 |
| --- | --- | --- |
| p value | 0.96 | |
| Median | 0 | 11 |
| --- | --- | --- |
| p value | 0.32 | |
| Median | 4 | 5 |
| --- | --- | --- |
| p value | 0.24 | |
(j)
(l)
(i)
(k)
| Median | 125 | 155 |
| --- | --- | --- |
| p value | 0.65 | |
| Median | 150 | 230 |
| --- | --- | --- |
| p value | <0.05 | |
| Median | 160 | 160 |
| --- | --- | --- |
| p value | 0.95 | |
| Median | 170 | 170 |
| --- | --- | --- |
| p value | 0.49 | |

## Slide 10
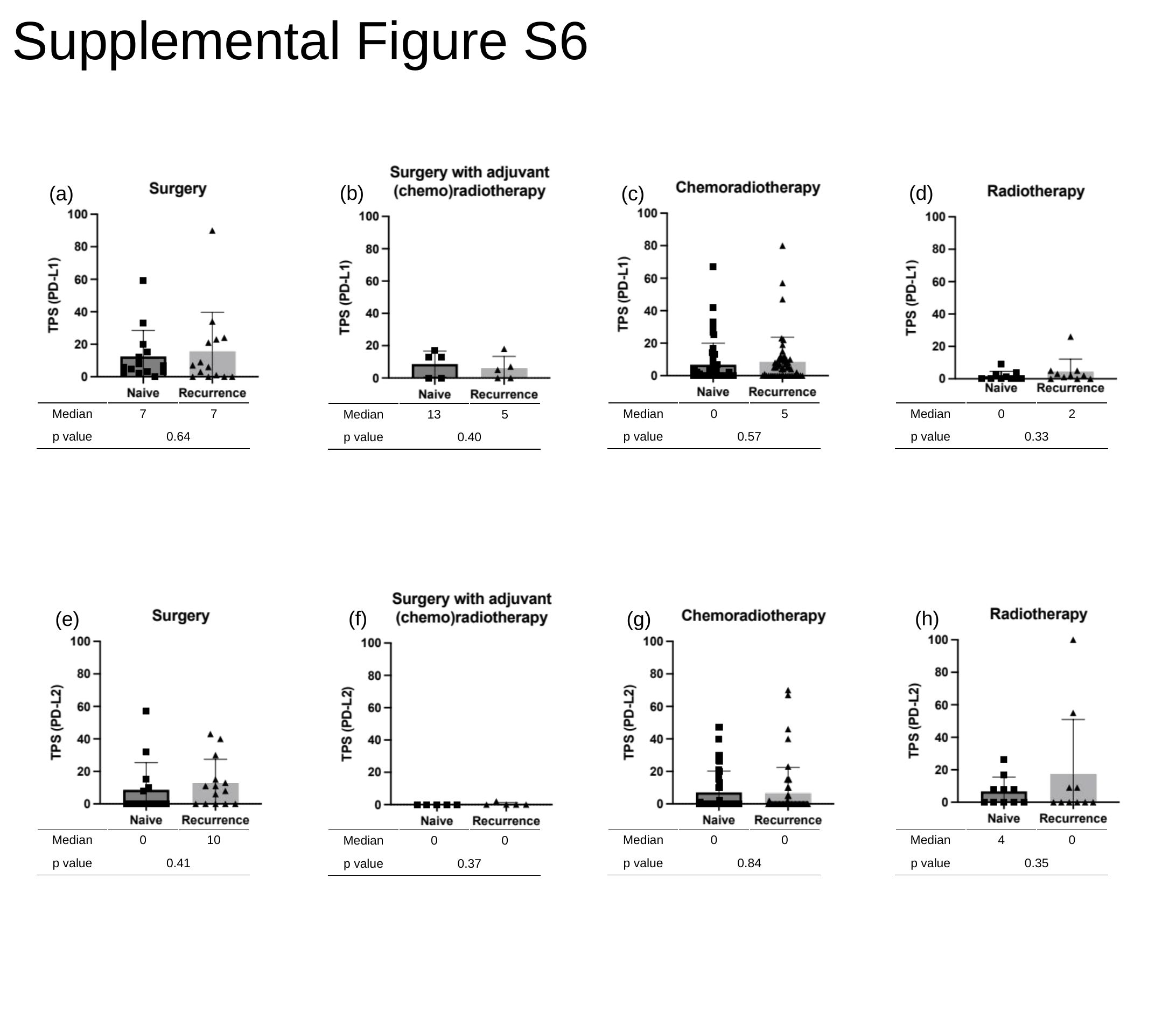

Supplemental Figure S6
(b)
(d)
(a)
(c)
| Median | 7 | 7 |
| --- | --- | --- |
| p value | 0.64 | |
| Median | 0 | 5 |
| --- | --- | --- |
| p value | 0.57 | |
| Median | 0 | 2 |
| --- | --- | --- |
| p value | 0.33 | |
| Median | 13 | 5 |
| --- | --- | --- |
| p value | 0.40 | |
(f)
(h)
(e)
(g)
| Median | 0 | 10 |
| --- | --- | --- |
| p value | 0.41 | |
| Median | 0 | 0 |
| --- | --- | --- |
| p value | 0.84 | |
| Median | 4 | 0 |
| --- | --- | --- |
| p value | 0.35 | |
| Median | 0 | 0 |
| --- | --- | --- |
| p value | 0.37 | |
